# Dissociated cerebellar contributions to feedforward gait adaptation

**DOI:** 10.1007/s00221-024-06840-9

**Published:** 2024-05-17

**Authors:** Karen L. Bunday, Toby J. Ellmers, M. Rashmi Wimalaratna, Luxme Nadarajah, Adolfo M. Bronstein

**Affiliations:** 1 Psychology, School of Social Sciences, University of Westminster, 115 New Cavendish Street, London, UK; 2 Centre for Vestibular Neurology, Department of Brain Sciences, Imperial College London, Charing Cross Hospital, London, UK

**Keywords:** Cerebellum, Cerebellar, Ataxia, Gait, Locomotion, Adaptation, After-effect, Downbeat nystagmus

## Abstract

The cerebellum is important for motor adaptation. Lesions to the vestibulo-cerebellum selectively cause gait ataxia. Here we investigate how such damage affects locomotor adaptation when performing the ‘broken escalator’ paradigm. Following an auditory cue, participants were required to step from the fixed surface onto a moving platform (akin to an airport travellator). The experiment included three conditions: 10 stationary (BEFORE), 15 moving (MOVING) and 10 stationary (AFTER) trials. We assessed both behavioural (gait approach velocity and trunk sway after stepping onto the moving platform) and neuromuscular outcomes (lower leg muscle activity, EMG). Unlike controls, cerebellar patients showed reduced after-effects (AFTER trials) with respect to gait approach velocity and leg EMG activity. However, patients with cerebellar damage maintain the ability to learn the trunk movement required to maximise stability *after* stepping onto the moving platform (i.e., reactive postural behaviours). Importantly, our findings reveal that these patients could even initiate these behaviours in a feedforward manner, leading to an after-effect. These findings reveal that the cerebellum is crucial for feedforward locomotor control, but that adaptive locomotor behaviours learned via feedback (i.e., reactive) mechanisms may be preserved following cerebellum damage.

**Supplementary Information:**

The online version contains supplementary material available at 10.1007/s00221-024-06840-9.

## Introduction

Locomotion is a learned behaviour that typically requires little explicit cognitive input (Clark 2015). Yet, humans often adapt locomotion when exposed to a novel, perturbing context or environment. Due to the lag in the transmission of sensory input that signals a loss of balance, proactive adaptations are often triggered prior to encountering the perturbation (Dakin and Bolton 2018) – particularly when the perturbation is too large to be counteracted by (comparatively slow) reactive postural adjustments alone (Dakin and Bolton 2018). Take, for instance, stepping onto a patch of ice. Rather than maintaining usual locomotion and relying on corrective responses once a slip occurs, it is much safer to adapt movement in a proactive, feedforward (i.e., predictive) manner in anticipation of the perturbation. As a result, the CNS must constantly monitor incoming sensory information for evidence of a likely future loss of balance, as doing so is crucial for the triggering of feedforward motor adaptations (Dakin and Bolton 2018). This is a process known as ‘motor adaptation’, whereby a well-learned movement is adjusted over a period of trial-and-error practice to account for new demands (Martin et al. 1996a; Bastian 2008). Adaptation can occur after just minutes or take many hours (Bastian 2008). Following adaptation, it is typically difficult to immediately return to baseline behaviour, leading to what is known as an ‘after-effect’, whereby erroneous behavioural adjustments remain and subsequently have to be de-adapted (Bastian 2008; Reisman et al. 2010).

The cerebellum plays a crucial role in feedforward motor adaptation (Bastian 2006; Pisotta and Molinari 2014; Therrien and Bastian 2019). The cerebellum, described as a ‘sensory predictor’, is hypothesised to generate estimations of the sensory consequences of movement (Therrien and Bastian 2019). The role of these predictions are two-fold. First, they allow the musculoskeletal system to successfully initiate the desired movement. But they are also used as a comparison against which to compare the *actual* sensory feedback received when performing the movement. Any resultant prediction errors (i.e., discrepancies between predicted and realised sensory consequences) are then used to update future predictions and further refine feedforward movement (Shadmehr et al. 2010).

Research has described how damage to the cerebellum can impair predictive feedforward adaptation of locomotion, whilst reactive control remains largely intact. For instance, Morton and Bastian (2006) asked participants to walk on a split-belt treadmill in which each belt was driven at different speeds; a paradigm which results in both reactive and predictive types of locomotor behaviour. Although both healthy controls and cerebellar patients exhibited reactive changes in stepping to contend with this speed difference, feedforward motor adaptations were limited in cerebellar patients (slower adaptations and reduced after-effects). Whilst this work provides insight on the cerebellum’s role in feedforward locomotion during split-belt walking, less is known about the cerebellum’s role during more commonly experienced perturbations such as stepping onto a slippery or unstable surface. In this previous work, treadmill speed was also matched across cerebellar patients and healthy controls. As such, the walking task may have presented an inherently greater challenge for patients – which may have influenced the results presented. Finally, although cerebellar patients *can* display intact reactive behavioural responses to simple perturbations (e.g., changes in split-belt treadmill speed) during walking, there is often considerable variability in the timing and duration of muscle activation patterns (Rand et al. 1998). We address these gaps by investigating kinematic and neuromuscular adaptation in patients with cerebellar lesions experiencing predictable balance perturbations whilst walking – with task difficulty adapted for individual gait disability. Unlike previous work which studied patients with diffuse cerebellar degeneration, we focused exclusively on patients with the Gait Ataxia Downbeat Nystagmus Syndrome, a condition which arises from damage to the vestibulo-cerebellum (Zee et al. 1976; Pierrot-Deseilligny and Milea 2005; Patel and Zee 2014).

Here, we investigate the role of the cerebellum in feedforward locomotion using the well-established ‘broken escalator’ paradigm (Reynolds and Bronstein 2003; Bunday et al. 2006; Bunday and Bronstein 2008; Bronstein et al. 2009; Lin et al. 2020). This involves participants repeatedly walking onto a moving surface, much like stepping onto an airport travellator. Although initially perturbing to postural stability, healthy controls soon adapt and learn to step onto this moving surface without losing their balance (Reynolds and Bronstein 2003; Bunday et al. 2006; Bunday and Bronstein 2008; Bronstein et al. 2009; Lin et al. 2020). Once the adaptation phase is complete, participants are then unequivocally warned that the surface will no longer move. This then causes a robust after-effect in healthy controls, characterised by increased gait velocity, increased activation of the lower-leg muscles, and a resultant stumble-like response (Reynolds and Bronstein 2003; Bunday et al. 2006; Bunday and Bronstein 2008; Bronstein et al. 2009; Lin et al. 2020). If the cerebellum plays a role in predictive locomotor adaptation (Morton and Bastian 2006; Pisotta and Molinari 2014), it is hypothesised that cerebellar patients (CBL) would demonstrate impairments during the post-adaptation phase of the broken escalator paradigm. Consequently, we predicted a reduced or absent locomotor after-effect in both kinematic and neuromuscular outcomes in this patient group.

## Methods

### Participants

Eight patients with the Gait Ataxia Downbeat Nystagmus Syndrome, aged 56–73 years (mean ± SD = 68 ± 7 years, female = 5/8 (62.5%)), were recruited. Twelve healthy controls matched closely in age and gender, aged 51-75yrs (mean ± SD = 64 ± 6 years, female = 7/12 (58.3%); no significant between-group difference in age (t_(17)_ = 1.35, *p* = .195)), were also tested. All controls were in good health with no history of neurological illness.

All patients underwent neurological examination by an experienced neurologist (AMB) to confirm downbeat nystagmus and gait ataxia but no upper limb ataxia, thus implicating vestibulo-cerebellar (flocculo-nodular-parafloccular) damage (Zee et al. 1976; Pierrot-Deseilligny and Milea 2005; Patel and Zee 2014). The nystagmus was seen by naked eye in all cases, enhancing on convergence, lateral gaze, head shaking and positional manoeuvres, typical for this syndrome (Wagner et al. 2008). The Gait Ataxia Downbeat Nystagmus Syndrome is caused by dysfunction of the vestibulo-cerebellum and is selectively reproduced in monkeys by flocculectomy (Zee et al. 1981). It is associated with pathology in the cranio-cervical junction (Bronstein et al. 1987) and functional imaging demonstrates changes in the cerebellar tonsils, flocculus and paraflocculus bilaterally (Dieterich and Brandt 2008). In our patients, the main aetiology was idiopathic (*n* = 7), with one patient with familial downbeat nystagmus, gait instability and a SCA6 phenotype, but who did not wish to undertake genetic testing. MRI scans were reported by consultant neuroradiologists as vermal atrophy (*n* = 2), superior cerebellar atrophy (*n* = 1), flocculo-nodular atrophy (*n* = 2) and within normal limits for age in the rest (*n* = 3). Gait ataxia Downbeat Nystagmus Syndrome is a well-defined and fairly homogenous albeit rare neurological syndrome, diagnosed in around only 2% of patients seen per year in highly specialised balance centres (Wagner et al. 2008). Given the nature of our task, patients with major gait disorder had to be excluded. The number of participants recruited (*n* = 8) is comparable to previous research investigating gait adaptation (which used *n* = 9 (Morton and Bastian 2006) and reaching adaptation (which used *n* = 7 or *n* = 9 (Tseng et al. 2007; Schlerf and Ivry 2011)) in patients with cerebellar damage.

Prior to testing, all participants completed an overground walking assessment (modified version of the equilibrium coordination test used to assess gait ataxia severity (Armutlu et al. 2001)) as an evaluation of gait ataxia. Participants were instructed to walk as fast as they comfortably could over 3 m whilst keeping their feet between two parallel lines placed 20 cm apart. Participants were timed as they walked up and down continuously three times. On average, the CBL participants stepped out of the designated walkway with 51.1% of their steps (SD = 16.1%, range = 35.0–68.3%) compared to the controls who only placed 2.2% (SD = 3.8%, range = 0–11.8%, 9/12 scoring 0%) of steps outside the designated lines (Z = -3.66, *p* < .001). Additionally, the patients took on average 22 ± 12 s to complete the task, whereas the controls only took 10 ± 2 s (Z = -3.56, *p* < .001).

The experimental procedure was approved by the Imperial College London ethics committee, and informed consent was obtained for all participants. Data collection took place in the Balance Lab at Charing Cross Hospital, London UK.

### Materials

**Fig. 1:**
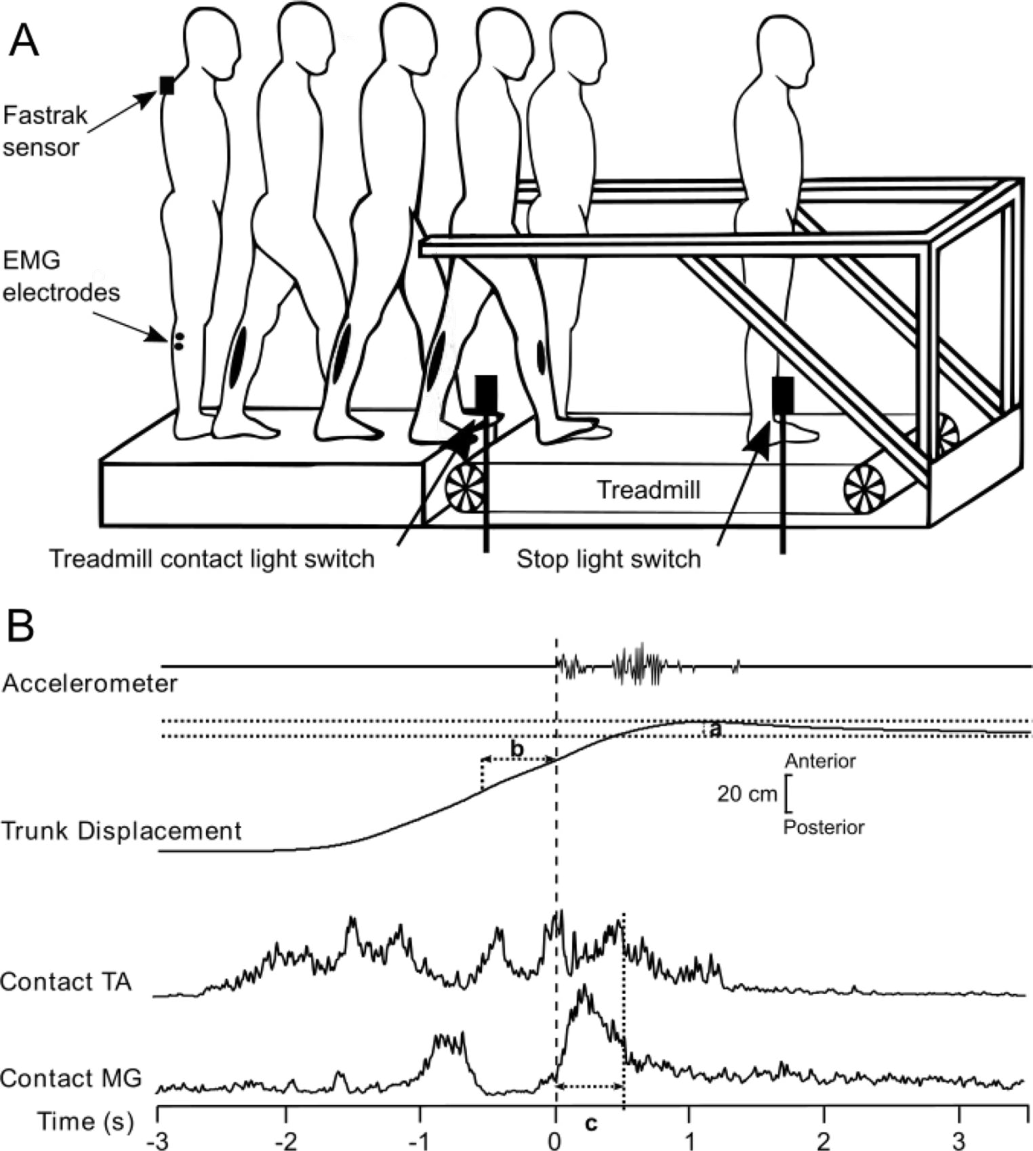
Experimental setup showing how participants walk from the fixed platform onto the treadmill (Fig. 1A). Participants take two steps on the fixed platform before stepping onto the treadmill with their third step. Note, the stop-light switches were used to deactivate the treadmill motor during MOVING trials. Figure 1B shows experimental data for a single control subject recorded during a single stationary AFTER trial (Fig. 1B). A Fastrak sensor was placed at the C7 vertebrae and recorded linear trunk position along the antero-posterior axis, used to calculate gait approach velocity and trunk sway, after stepping onto the treadmill (Fig. 1B). Electomyographic (EMG) electrodes recorded muscle activity of the medial gastrocnemius (MG) and tibialis anterior (TA) for the leg that stepped onto the treadmill (Fig. 1B).

A standard motorised treadmill (HP Cosmos Mercury Med v4.0 LT, Germany), with a running surface/belt of 150 cm x 50 cm, housed specialised handrails (height: 105 cm) that extended 60 cm beyond the end of the treadmill (Fig. 1A). The belt movement direction was reversed to carry participants in the forward direction. A fixed platform, 142 cm in length, stood at the foot of the treadmill where the handrails overhung (Fig. 1A). A computer programme controlled the treadmill speed. Belt movement was initiated by an additional specialised programme (‘Acquire’, D. Buckwell, MRC/Imperial College London) which in turn recorded all movement data (see below). Two infrared light switches were placed along the treadmill: The first, at the beginning of the belt, marking when participants passed from the fixed platform to the treadmill (Fig. 1A), and the second, placed towards the end of the belt, made the treadmill belt stop.

Sagittal trunk position was measured using a Fastrak electromagnetic tracking device (Polhemus, VT, USA). The sensor was placed over the C7 vertebrae, and the transmitter was suspended over the foot of the treadmill (Fig. 1A). Step timing information was provided by footswitches (Flexiforce, Teckscan, MA, USA) placed under the first metatarsal phalangeal joint and heel. A treadmill-mounted accelerometer (Entran, Watford, UK) provided independent, accurate foot-treadmill contact timing. EMG from the medial gastrocnemius (MG) and tibialis anterior (TA) muscles of each leg was recorded. These muscles were selected due to previous research highlighting their involvement in stabilisation following foot contact onto the treadmill (Reynolds and Bronstein 2003; Bunday and Bronstein 2009). Bipolar active surface electrodes (model DE2.1, Delsys Inc, USA) were placed on the belly of the muscle. The EMG signals were amplified and filtered (20–1000 Hz; Bagnoli 16, Delsys Inc, USA). EMG and kinematic data were sampled at 2000 Hz and recorded by a specialised computer programme (‘Acquire’, D. Buckwell, MRC/Imperial College London) for off-line analysis.

### Procedure

The broken escalator paradigm consisted of 3 successive conditions: BEFORE, MOVING and AFTER. All participants completed ten baseline (BEFORE) trials. Gait was initiated by a recorded verbal “WALK” auditory cue; participants took two steps, starting with their left or right foot whichever was dominant, on the fixed platform before stepping onto the stationary treadmill with their third step. Here they adopted a quiet stance until recording had finished. Each trial lasted for 16 s. Participants then completed 15 MOVING trials where they stepped on the moving treadmill. Individual treadmill speed was set at 40% higher than the participant’s preferred gait speed when stepping onto the stationary treadmill (average of 3 trials). This customisation ensured that the postural perturbation (i.e. ‘error signal’) experienced when stepping onto the moving treadmill was comparable across groups; given that the larger discrepancy between gait approach velocity and the velocity of the treadmill, the larger the postural instability (Bronstein et al. 2009). However, to ensure that such normalisation did not contribute to any differences observed, additional statistical analyses were conducted whilst controlling for treadmill speed (see Statistical Analysis section below). On average, the CBL and Control preferred walking speeds were 0.55 ± 0.20 m/s and 0.71 ± 0.11 m/s (Z = -1.54, *p* = .123), respectively. Therefore, customs treadmill speeds were 0.72 ± 0.28 m/s for the CBL group and 0.99 ± 0.12 m/s for the Control group (Z = -1.54, *p* = .123).

During the MOVING condition, participants could see the treadmill initiate the running belt. They were instructed not to use the handrails unless necessary when stepping onto the treadmill. Patients who required assistance were further supported (usually during the first and second MOVING trials only) by two experimenters who stood either side of the treadmill. Having completed the MOVING trials, a clear and unequivocal verbal warning that the treadmill belt would no longer move was given. The treadmill belt was taped down for visual affirmation and stability. Participants then completed ten stationary AFTER trials.

### Data analysis

Pre- and post-foot-treadmill contact time points were derived from the treadmill-mounted accelerometer and corroborated with the infrared switch and foot switch data (Fig. 1B). For stationary trials (BEFORE and AFTER), trunk sway was defined as the maximum forward deviation (‘overshoot’) of the trunk, relative to the mean final resting stance position in the last 3 s of the trial, from the Fastrak linear trunk position trace (Fig. 1B(a)). Due to the nature of the trunk sway data, the same criteria could not be used during the MOVING trials; thus, this data was omitted from the results. The velocity at which participants approached the treadmill was calculated using the position sensor on the trunk, defined as the mean gait approach velocity in a 0.5 s time window before foot-treadmill contact (Fig. 1B(b)) (Bunday and Bronstein 2008). Significant EMG bursts that propelled the participants onto the treadmill and stabilised them once contact was made with the treadmill were measured. Contact leg (i.e., the first leg to contact the treadmill) EMG activity (TA and MG) were rectified and integrated in a 500 ms window post foot-treadmill contact. Data for the MG of the contact leg was discarded for one control participant due to poor data quality. Data were provided a randomised code and analysis was conducted blinded to group status.

### Statistical analysis

Data were averaged across BEFORE trials (3–10) to produce a mean BEFORE value for each variable. The first 2 trials were discarded as these trials were used to ensure familiarisation with the apparatus and general experimental procedure. Further discarding of the first two trials in the other conditions were not deemed necessary because apparatus and instructions did not change but also because the locomotor after-effect we investigate is only prominent in the first AFTER trial (Tang et al. 2013).

First, we used Mann-Whitney *U* tests to compare between-group differences for each outcome variable during BEFORE trials. As most outcome variables were non-normally distributed, comparisons between different conditions were analysed using a Generalised Estimating Equation (GEE). We chose an exchangeable working correlation matrix to define dependency among measurements. We ran a separate GEE for each individual variable (trunk sway, gait approach velocity and EMG). MOVING (i.e., adaptation) and AFTER (i.e., ‘de-adaptation’ or after-effect) trials were analysed separately. For MOVING trials, we conducted two separate analyses. We first compared BEFORE to MOVING trial 1 (2 group levels (CBL and Controls) and 2 trial levels (BEFORE and MOVING-1), and then compared MOVING trial 1 to MOVING trial 15 (2 group levels (CBL and Controls) and 2 trial levels (MOVING-1 and MOVING-15). This allowed us to define the presence of locomotor adaptation. The presence of an after-effect was determined by analysing factors group (2 levels: CBL and Controls) and trial (2 levels: BEFORE and first AFTER). A significant difference between BEFORE and first AFTER would provide evidence of a locomotor after-effect (Reynolds and Bronstein 2003; Bunday and Bronstein 2008; Bronstein et al. 2009). For all GEE analyses, Bonferroni corrected post-hoc *t*-tests followed up significant effects. To ensure that any findings observed were not a direct consequence of the cerebellar patients encountering a (non-significant) slower treadmill speed, all analyses were then repeated whilst controlling for individual treadmill speed.

## Results

**Fig. 2:**
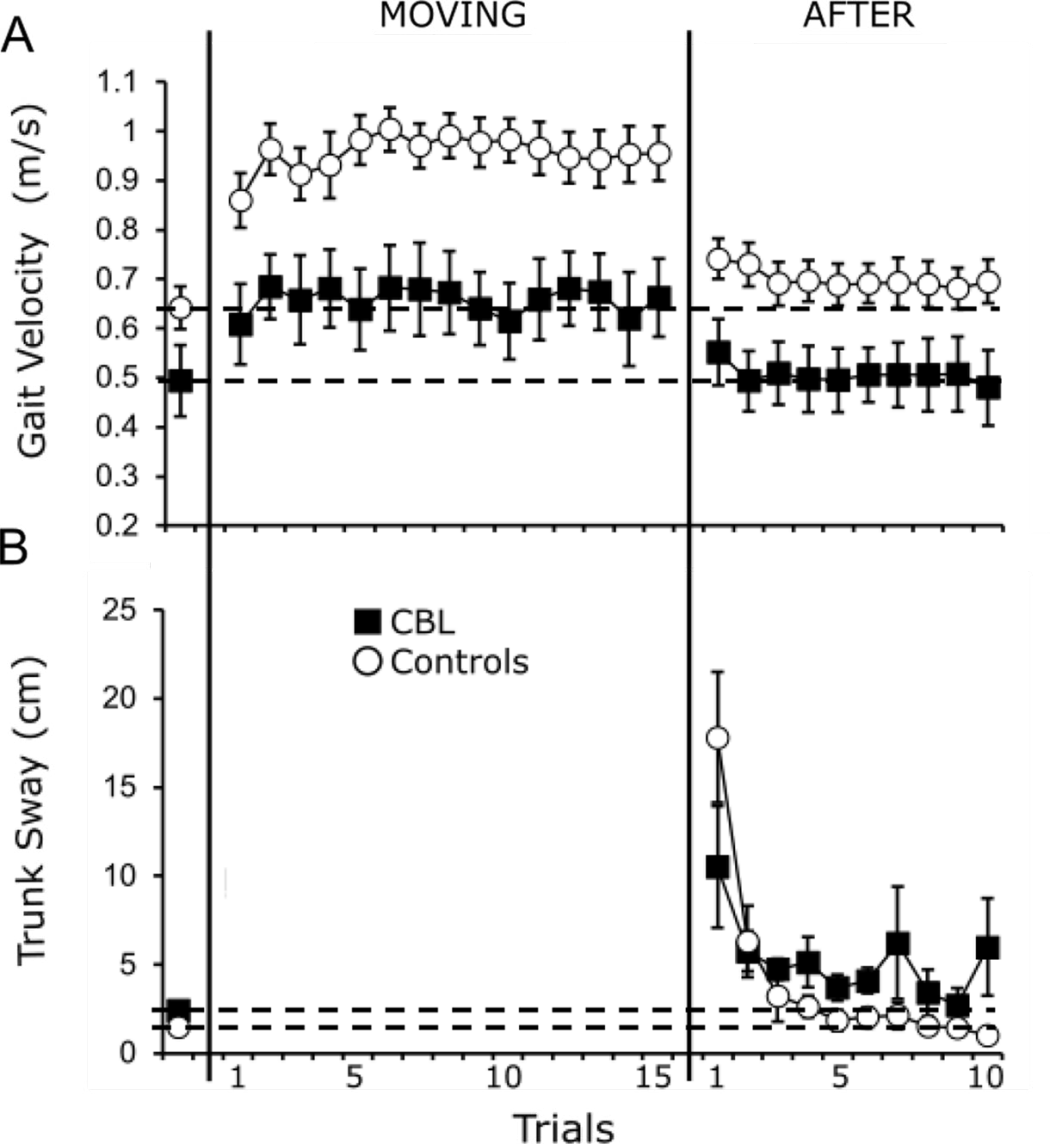
Group kinematic data (mean ± SE) during mean BEFORE (left panel), MOVING (middle panel; gait velocity only) and AFTER (right panel) conditions for gait approach velocity (Fig. 2A) and trunk sway (Fig. 2B). Numbers along the x-axis represent trial numbers. Group data for the cerebellar patients (CBL; closed squares) and controls (white circles) are represented. Note, due to the very low SE values for trunk sway in BEFORE trials (for both groups), SE bars do not extend beyond the outer-limits of the squares/circles during BEFORE. Due to the criteria used to calculate trunk overshoot, it is not possible to calculate this outcome during MOVING trials and hence these data are omitted from analysis

**Fig. 3:**
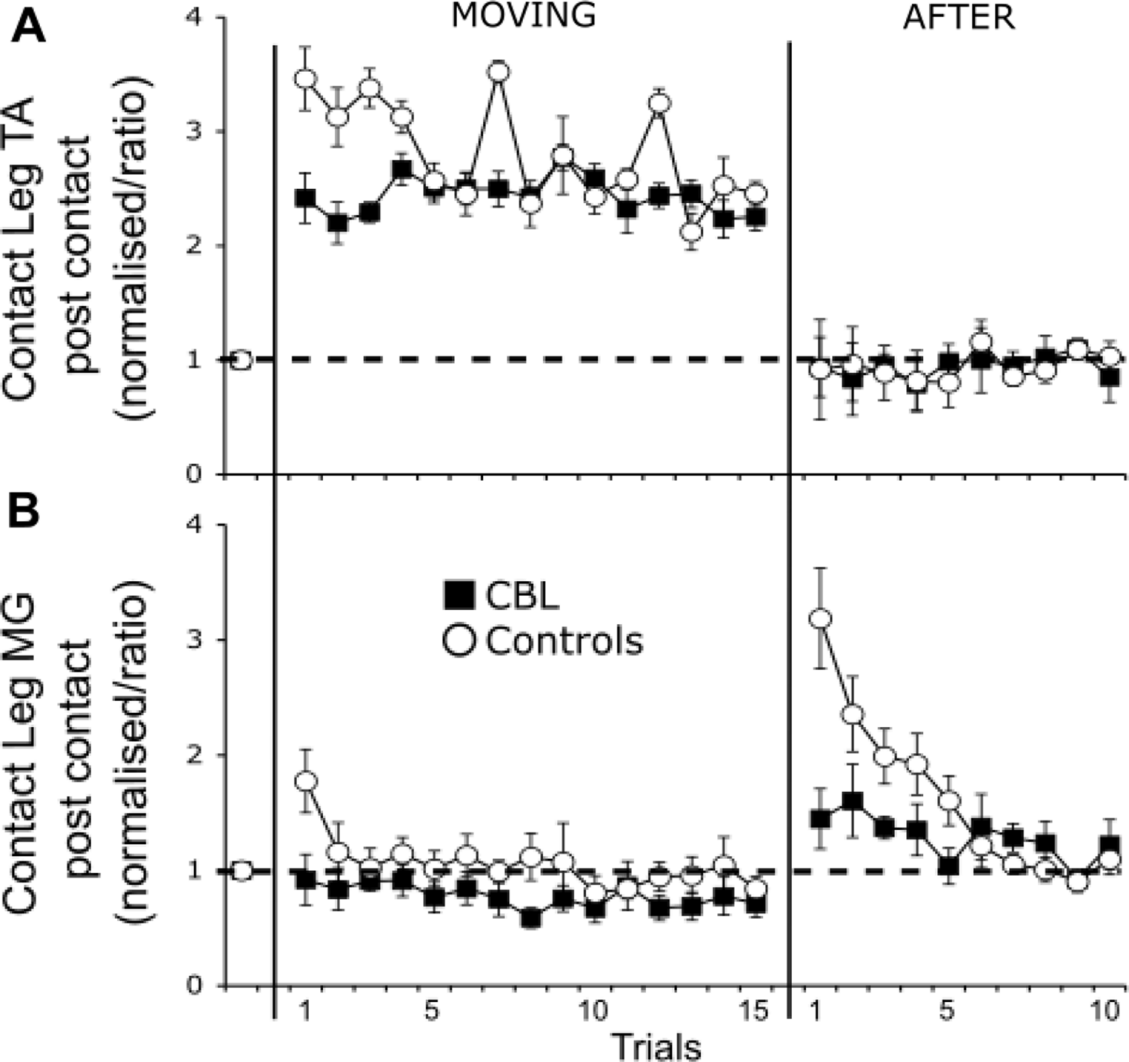
Normalised (to mean BEFORE) group electromyographic (EMG) data (mean ± SE) during MOVING and AFTER conditions for the contact leg tibialis anterior (TA; Fig. 3A) and medial gastrocnemius (MG; 3B). Numbers along the x-axis represent trial numbers. Group data for the cerebellar patients (CBL; closed squares) and controls (white circles) are represented. The lack of an EMG aftereffect in the patients is best seen in the contact leg MG.

**Fig. 4:**
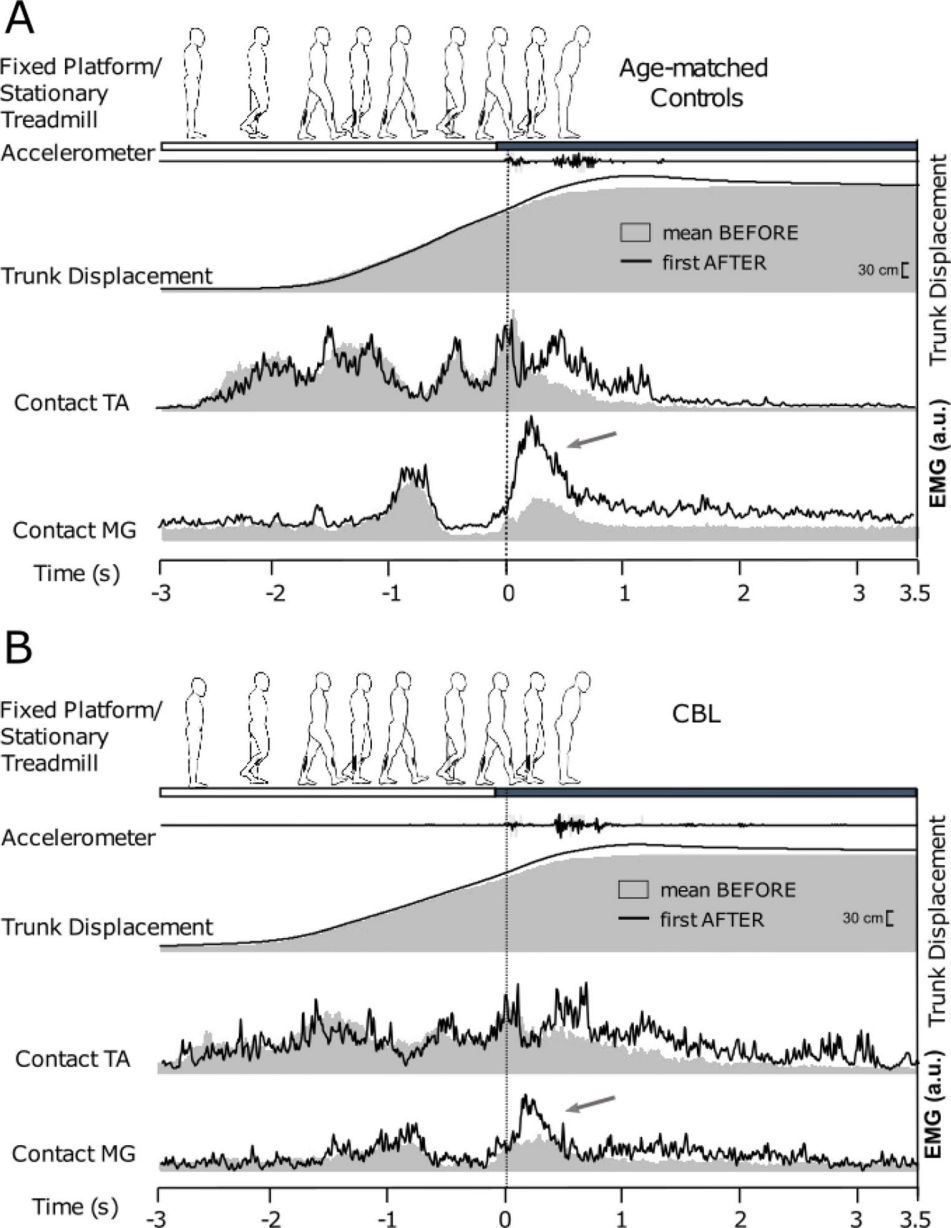
Grand averaged of control (Fig. 4A) and cerebellar patients’ (CBL; Fig. 4B) data of movement and EMG responses during mean BEFORE (grey shaded area) and first AFTER trial (i.e., the aftereffect; solid line). The blank area delineated between the solid line and the grey area is a visual representation of the aftereffect magnitude – note the smaller ‘blank’ area (see arrows) for the CBL group as compared to the control subjects with respect to the contact leg medial gastrocnemius (MG) electromyographic (EMG) activity.

Mean (± SE) values for kinematic and EMG during BEFORE, MOVING and AFTER trials are presented in Figs. 2 and 3, respectively. Grand averages for all outcomes for mean BEFORE and the first AFTER trial are presented in Fig. 4A (Control group) and 4B (CBL group).

### Before comparisons

Whilst gait approach velocity tended to be lower in the CBL patients during BEFORE trials, this difference was not statistically significant (*Z*=-1.54, *p* = .123). CBL patients did, however, have statistically greater trunk sway post foot contact during BEFORE trials (*Z*=-2.32, *p* = .021) – indicating greater postural instability following gait termination.

Regarding EMG, there were no statistically significant between-group differences for either contact leg MG (*Z*=-0.91, *p* = .364) or TA activity (*Z*=-1.31, *p* = .190).

### Before-to-moving comparisons – gait approach velocity

There was a clear pattern of increased gait approach velocity during the first MOVING trial for Control participants (mean 33.4% increase), whilst the CBL group showed increases of around half this magnitude (mean 15.4% increase). Indeed, whilst there was a significant main effect of both group (*χ*^*2*^ *=* 5.87, *p* = .015) and trial (*χ*^*2*^ *=* 41.16, *p* < .001), the significant interaction effect (*χ*^*2*^ *=* 7.21, *p* = .007) and subsequent Bonferroni post-hocs revealed that the increase in gait velocity from BEFORE to MOVING-1 was significant for the Control group only (*p* < .001; CBL group, *p* = .104). These results remained when controlling for treadmill speed (see Supplementary Materials).

### Before-to-moving comparisons – EMG

With respect to contact leg TA (the muscle activity required to arrest a backwards fall after stepping onto the moving treadmill), both groups showed a significant increase from BEFORE to MOVING-1, although this increase tended to be larger for the Control group: There was a significant main effect of trial (*χ*^*2*^ *=* 61.58, *p* < .001), a near significant effect of group (*χ*^*2*^ *=* 3.76, *p* = .052), and a significant interaction between the two (*χ*^*2*^ *=* 5.32, *p* = .021). Post-hoc tests revealed that EMG activity in contact leg TA significantly increased from BEFORE to MOVING-1 in both groups (Control: *p* < .001, CBL: *p* = .002). Whilst the increase in contact leg TA activity tended to be larger for the Control group, this was not statistically significant (*p* = .194). Due to the forwards-moving treadmill causing participants to fall backwards, the MG has a reduced role in maximising postural stability during MOVING trials. Indeed, there was limited significant change between BEFORE and MOVING-1 for either group with respect to contact leg MG activity: There was neither a significant main effect of group (*χ*^*2*^ *=* 0.84, *p* = .772) nor trial (*χ*^*2*^ *=* 1.91, *p* = .167), nor a significant interaction (*χ*^*2*^ *=* 1.51, *p* = .220).

When controlling for treadmill speed, the key patterns of results remained; however, the increase in contact leg TA activity during MOVING-1 was now significantly larger for the Control group (*p* = .047), suggesting an impairment in reactive neuromuscular control for patients with cerebellar damage. Please see Supplementary Materials for the full results from the covariate analysis.

### Early-to-late Moving ‘adaptation’ trials – gait approach velocity

There was a main effect of both group (*χ*^*2*^ *=* 9.80, *p* = .002) and trial (*χ*^*2*^ *=* 5.37, *p* = .020) for gait velocity during the MOVING trials, but no significant interaction effect (*χ*^*2*^ *=* 0.46, *p* = .496). Gait speed was significantly greater for the Control group throughout (i.e. patients walk slower), and gait speed also significantly increased from MOVING-1 to MOVING-15, irrespective of the group. This indicates that both Control and CBL group were able to adapt and increase their gait speed during subsequent MOVING trials. These results remained when controlling for treadmill speed (see Supplementary Materials).

### Early-to-late moving ‘adaptation’ trials – EMG

EMG data revealed evidence of across-trial neuromuscular adaptation for both groups. For contact leg MG, there was a significant main effect of trial during MOVING trials (*χ*^*2*^ *=* 16.29, *p* < .001); with reduced EMG activity during MOVING-15 compared to MOVING-1. There was no significant effect of group (*χ*^*2*^ *=* 0.36, *p* = .551), nor a significant interaction (*χ*^*2*^ *=* 1.09, *p* = .297); indicating that EMG activity in the contact leg MG decreased across MOVING trials irrespective of group. For contact leg TA, whilst there was no significant main effect of group (*χ*^*2*^ *=* 2.30, *p* = .130), there was a significant main effect of trial (*χ*^*2*^ *=* 7.54, *p* = .006) and a significant interaction between the two (*χ*^*2*^ *=* 4.36, *p* = .037). Post-hoc tests revealed a non-significant decrease in contact TA (the more strongly activated muscle during MOVING trials) from MOVING-1 to MOVING-15 for Control group (*p* = .078), but no change for the CBL group (*p* = 1.00) – indicating reduced adaptation. These results remained when controlling for treadmill speed (see Supplementary Materials).

### After ‘de-adaptation’ trials – kinematic outcomes

As illustrated in Fig. 2, there was a general pattern of consistent locomotor after-effects in both kinematic variables for the control participants during the first AFTER trial (mean increase of 0.1 m/s for gait approach velocity and 16.36 cm for trunk sway with respect to baseline, respectively). In comparison, CBL patients tended to only exhibit after-effects for trunk sway (mean increase of 7.38 cm) – a reactive response (learned during MOVING trials to maximise postural stability) that is subsequently triggered in a feedforward ‘pre-emptive’ manner (Tang et al. 2013). In contrast, they exhibited limited after-effects for gait approach velocity (mean increase of 0.03 m/s), which reflects a true feedforward behaviour.

With respect to gait approach velocity during the first AFTER trial, there was a main effect of both group (*χ*^*2*^ *=* 5.16, *p* = .023) and trial (*χ*^*2*^ *=* 15.02, *p* < .001), and a significant interaction (*χ*^*2*^ *=* 3.88, *p* = .049). Bonferonni corrected post-hocs revealed a significant after-effect (i.e., significant increase in velocity in AFTER-1 compared to BEFORE) for the Control group (*p* < .001), but not the CBL group (*p* = .943). Whilst there was no main effect of group with respect to trunk sway (*χ*^*2*^ = 1.46, *p* = .227), there was a significant main effect of trial (*χ*^*2*^ = 21.99, *p* < .001). The interaction effect was not significant (*χ*^*2*^ = 3.15, *p* = .076), indicating that trunk sway was significantly greater during AFTER-1 compared to BEFORE trials for both groups. These results remained when controlling for treadmill speed (see Supplementary Materials).

### After ‘de-adaptation’ trial – EMG

Figure 4 shows the grand averaged trunk displacement and EMG in both CBL and Control group. Mean BEFORE (grey shaded area/line) and first AFTER (solid line) are shown for both groups. The EMG shown up to heel contact with the treadmill (-3–0 s) corresponds to gait initiation and 1.5 gait cycles (i.e., 3 steps with the 3rd resulting in resting stance on the treadmill).

The general pattern for neuromuscular control outcomes revealed consistent after-effects for ‘braking’ MG activity for the Control group; and a general absence of any significant neuromuscular after-effects for the CBL group. At the point of foot-contact onto the treadmill, the Control group display a clear increase in contact MG activity in first AFTER compared to baseline EMG (Fig. 4A). This burst constitutes increased gait termination (‘braking’) activity as part of the locomotor after-effect that is triggered in an anticipatory manner prior to foot-contact (Reynolds and Bronstein 2003; Bunday and Bronstein 2008; Bronstein et al. 2009). This MG burst is significantly reduced (oblique arrow) and delayed (vertical arrow) in the patients (Fig. 4B; compare with control data in Fig. 4A). Indeed, with respect to contact leg ‘braking’ MG activity during the first AFTER trial; whilst there was no significant main effect of group (*χ*^*2*^ *=* 1.70, *p* = .192), there was both a significant effect of trial (*χ*^*2*^ *=* 32.06, *p* < .001) and also a significant interaction effect (*χ*^*2*^ *=* 9.16, *p* = .002). Bonferonni corrected post-hocs revealed a significant after-effect (i.e., significant increase in contact leg MG activity 500 ms after foot-contact during AFTER-1 compared to BEFORE) for the Control group (*p* < .001), but not the CBL group (*p* = .190). For contact leg TA, there was neither a significant main effect of group (*χ*^*2*^ *=* 0.98, *p* = .323) nor trial (*χ*^*2*^ *=* 3.22, *p* = .073), nor a significant interaction between the two (*χ*^*2*^ *=* 0.40, *p* = .529). Combined, these results reveal a general absence of after-effects for the CBL group, with respect to neuromuscular variables. These results remained when controlling for treadmill speed (see Supplementary Materials).

## Discussion

The present work investigated the role of the cerebellum when adapting to a locomotion task that reflects a commonly encountered activity of daily living: stepping onto a moving surface, such as a travellator or escalator. Our results reveal that individuals with cerebellar damage (Gait Ataxia Downbeat Nystagmus syndrome) display reduced after-effects in both kinematic (gait approach velocity) and electro-muscular (‘braking’ EMG activity) locomotor outcomes. This provides further evidence of the importance of the cerebellum in feedforward locomotor adaptation, partly supporting the previous study on this topic (Morton and Bastian 2006). However, unlike previous work (Morton and Bastian 2006), we discovered that cerebellar patients showed some degree of feedforward adaptation; specifically, with respect to trunk sway (i.e., reactive postural behaviours), resulting in an overall significant after-effect for this variable. These findings highlight dissociated cerebellar contributions to feedforward gait adaptation.

### Reduced – but not absent – locomotor after-effects in cerebellar patients

A previous paper has described how cerebellar damage impairs the ability to make feedforward locomotor adaptations, with limited after-effects observed for cerebellar patients compared to healthy controls (Morton and Bastian 2006). This supports work which describes generally impaired feedforward adaptation following cerebellar damage in a range of other tasks, including those requiring upper limb or saccadic eye movements (Martin et al. 1996b; Krupa and Thompson 1997; Lang and Bastian 1999, 2001; Smith and Shadmehr 2005; Xu-Wilson et al. 2009; Pisotta and Molinari 2014). Broadly speaking, our results support this previous work. Whilst the Control group exhibited a robust after-effect for gait approach velocity during the first AFTER trial (mean 15.6% increase compared to BEFORE), the CBL group exhibited limited and non-significant after-effects (mean increase 5.8%).

In contrast, both CBL patients and controls showed significant after-effects with respect to trunk sway (the trunk overshoot experienced *after* stepping onto the now stationary treadmill). Although the after-effect tended to be somewhat smaller in the patients, the present findings reveal that individuals with cerebellar damage are able – to some degree, at least – to trigger certain locomotor behaviours in a feedforward manner, resulting in an observed after-effect. A previous paper has described how cerebellar damage impairs the ability to make feedforward locomotor adaptations, whilst the ability to make rapid reactive locomotor adjustments remains relatively intact (Morton and Bastian 2006). Indeed, short-term reactive behaviours and longer-term adaptations appear to be controlled by different neural structures, with the latter being dependent upon the cerebellum (Markov et al. 2021). However, our findings imply that patients with cerebellar damage *can* also use longer-term feedforward mechanisms to trigger originally reactive, feedback-driven postural behaviours which results in the trunk sway locomotor after-effect observed. The ability to adapt reactive postural behaviours is similar to what has been shown previously in healthy controls, with respect to the adaptation of feedback-driven reaching movements (Cluff and Scott 2013). In the present paradigm, the trunk sway after-effect occurs as participants learn during the MOVING trials that they need to move their trunk (i.e., centre of mass) rapidly forwards to maintain their balance upon stepping onto the moving treadmill, as the treadmill motion propels their centre of mass backwards. In other words, it is a *reactive* behavioural adjustment that is triggered (during the AFTER trials) in a *feedforward* manner, leading to an after-effect (described previously with a similar paradigm as a ‘pre-emptive postural adjustment’ (Tang et al. 2013)).

In contrast to the trunk sway behaviour, gait approach velocity in the present paradigm reflects a purely feedforward, predictive locomotor behaviour – given that this requires that the individual anticipates the behavioural adjustments required ahead of time. We hypothesise that the after-effects observed for ‘reactive’ balance behaviours may be a consequence of the higher-level extra-cerebellar areas involved in processing, coordinating and adapting to postural responses following a loss of balance (Marlin et al. 2014; Mierau et al. 2015; Patel et al. 2019). In contrast, adapting one’s gait approach (i.e., acceleration) velocity in a feedforward manner during the present study requires accurate perception of both external visual motion (the moving surface/platform speed) and internal (visual and vestibular) self-motion. Both the former (Händel et al. 2009; Baumann et al. 2015) and the latter (Bronstein et al. 2008) have been shown to be impaired in patients with cerebellar lesions.

### Cerebellar damage impairs neuromuscular adaptations

Despite patients with cerebellar damage displaying some degree of feedforward locomotor adaptation (as evidenced by the significant after-effect in trunk sway), this was accompanied by a general absence of after-effects with respect to the assessed neuromuscular control outcomes. Prior research has shown that the forward trunk sway observed as part of the after-effect in this paradigm is counteracted by feed-forward mechanisms, with participants triggering ‘braking’ EMG of contact leg MG prior to the foot contacting the treadmill (Reynolds and Bronstein 2003; Bunday and Bronstein 2008; Bronstein et al. 2009). As illustrated in the grand averages presented in Fig. 4, the Control group exhibited large, anticipatory increases in this ‘braking’ muscular activity during the first AFTER trial – with this activity starting at the point of foot contact (“latency 0ms”). In other words, healthy controls *anticipated* (wrongly) that the surface would still be moving and triggered the muscular response required to maintain stability in a feedforward manner. In contrast, these anticipatory EMG bursts are reduced and delayed for the patients. This lack or reduction of the anticipatory EMG bursts represents the underlying neurophysiological correlate for the reduced feedforward postural behaviour in the patients. Further, although Controls demonstrated some evidence of adaptation for TA activity during MOVING trials (the most ‘activated’ muscle, required to arrest the backwards fall after stepping onto the forward-moving treadmill), no such change in TA activity was observed for the CBL group during MOVING trials. These findings extend previous research conducted during upper-limb tasks and provides evidence of the cerebellum’s role in generating anticipatory muscular activity during lower limb locomotor behaviour (Lang and Bastian 1999).

## Conclusion

In conclusion, these findings provide further evidence that the cerebellum plays a key role in feedforward adaptation, in this case in patients with locomotor disorder due to damage to the vestibulo-cerebellum. Despite the individual customisation of gait velocity in our experiment, these patients had difficulty initiating changes to gait speed or EMG activity in a feedforward manner (resulting in limited after-effects for gait velocity or EMG outcomes). However, the findings highlight dissociated cerebellar contributions to feedforward gait adaptation: Patients were able to learn reactive postural behaviours and initiate these in a feedforward manner, leading to observable after-effects in trunk sway. These findings reveal that the cerebellum is crucial for feedforward locomotor control, but that adaptive locomotor behaviours learned via feedback (i.e., reactive) mechanisms might be preserved following cerebellum damage. Clinical rehabilitation focus could therefore look to develop strategies that both maximise reactive control mechanisms in this population and enhance feedforward locomotor control.

### Electronic supplementary material

Below is the link to the electronic supplementary material.

Supplementary Material 1

## Data Availability

All analysed data can be freely accessed via an Open Science Framework repository (https://osf.io/gyuef/).
