# Supplementary material for: Dissociated cerebellar contributions to feedforward gait adaptation
Source: Exp Brain Res. 2024 May 17;242(7):1583–93. doi: 10.1007/s00221-024-06840-9 (PMC11208272; doi:10.1007/s00221-024-06840-9)
Supplement: Supplementary file 1 — Supplementary Material 1 [file 221_2024_6840_MOESM1_ESM.docx]

***BEFORE-to-MOVING comparisons – Gait approach velocity***

There was a significant main effect of both group (χ^2^*=*8.96, *p*=.003) and trial (χ^2^*=*41.16, *p*<.001). However, the significant interaction effect (χ^2^*=*7.21, *p*=.007) and subsequent Bonferroni post-hocs revealed that the increase in gait velocity from BEFORE to MOVING-1 was significant for the Control group only (*p*<.001; CBL group, *p*=.104). This resulted in gait approach velocity being significantly greater for the Control group during MOVING-1 compared to the CBL group (*p* = .009). The treadmill speed covariate was not significant (χ^2^*=*1.71, *p*=.191).

***BEFORE-to-MOVING comparisons – EMG***

There was a significant main effect of group (χ^2^*=*6.64, *p*=.010) and trial (χ^2^*=*61.58, *p*<.001), and a significant interaction between the two (χ^2^*=*5.32, *p*=.021). Post-hoc tests revealed that EMG activity in contact leg TA significantly increased from BEFORE to MOVING-1 in both groups (Control: *p*<.001, CBL: *p*=.002), with this increase in contact leg TA activity being significantly larger for the Control group (*p*=.047). The treadmill speed covariate was not significant (χ^2^*=*2.38, *p*=.123). In contrast, there was limited significant change between BEFORE and MOVING-1 for either group with respect to contact leg MG activity: There was neither a significant main effect of group (χ^2^*=*0.56, *p*=.455) nor trial (χ^2^*=*1.86, *p*=.173), nor a significant interaction (χ^2^*=*1.57, *p*=.210). The treadmill speed covariate was not significant (χ^2^*=*3.66, *p*=.056).

***Early-to-late MOVING ‘adaptation’ trials – Gait approach velocity***

There was a main effect of both group (χ^2^*=*12.23, *p*<.001) and trial (χ^2^*=*5.37, *p*=.020) for gait velocity during the MOVING trials, but no significant interaction effect (χ^2^*=*0.46, *p*=.496). Gait speed was significantly greater for the Control group throughout (i.e. patients walk slower), and gait speed also significantly increased from MOVING-1 to MOVING-15, irrespective of the group. The treadmill speed covariate was not significant (χ^2^*=*2.13, *p*=.144).

***Early-to-late MOVING ‘adaptation’ trials – EMG***

For contact leg MG, there was a significant main effect of trial during MOVING trials (χ^2^*=*16.18, *p*<.001); with reduced EMG activity during MOVING-15 compared to MOVING-1. There was no significant effect of group (χ^2^*=*0.95, *p*=.330), nor a significant interaction (χ^2^*=*1.13, *p*=.287); indicating that EMG activity in the contact leg MG decreased across MOVING trials irrespective of group. The treadmill speed covariate was not significant (χ^2^*=*2.140, *p*=.122). For contact leg TA, whilst there was no significant main effect of group (χ^2^*=*3.30, *p*=.069), there was a significant main effect of trial (χ^2^*=*7.54, *p*=.006) and a significant interaction between the two (χ^2^*=*4.36, *p*=.037). Post-hoc tests revealed a non-significant decrease in contact TA (the more strongly activated muscle during MOVING trials) from MOVING-1 to MOVING-15 for Control group (*p*=.078), but no change for the CBL group (*p*=.903). The treadmill speed covariate was not significant (χ^2^*=*0.94, *p*=.332).

***AFTER ‘de-adaptation’ trials – Kinematic outcomes***

With respect to gait approach velocity during the first AFTER trial, there was a main effect of both group (χ^2^*=*8.27, *p*=.004) and trial (χ^2^*=*15.02, *p*<.001), and a significant interaction (χ^2^*=*3.88, *p*=.049). Bonferonni corrected post-hocs revealed a significant after-effect (i.e., significant increase in velocity in AFTER-1 compared to BEFORE) for the Control group (*p*<.001), but not the CBL group (*p*=.943). The treadmill speed covariate was not significant (χ^2^*=*2.82, *p*=.093). Whilst there was no main effect of group with respect to trunk sway (χ^2^=1.19, *p*=.275), there was a significant main effect of trial (χ^2^=21.99, *p*<.001). The interaction effect was not significant (χ^2^=3.15, *p*=.076), indicating that trunk sway was significantly greater during AFTER-1 compared to BEFORE trials for both groups. The treadmill speed covariate was not significant (χ^2^*=*0.01, *p*=.943).

***AFTER ‘de-adaptation’ trial – EMG***

Whilst there was no significant main effect of group with respect to contact leg ‘braking’ MG activity during the first AFTER trial (χ^2^*=*2.173, *p*=.140), there was both a significant effect of trial (χ^2^*=*32.06, *p*<.001) and also a significant interaction effect (χ^2^*=*9.16, *p*=.002). Bonferonni corrected post-hocs revealed a significant after-effect (i.e., significant increase in contact leg MG activity 500 ms after foot-contact during AFTER-1 compared to BEFORE) for the Control group (*p*<.001), but not the CBL group (*p*=.190). The treadmill speed covariate was not significant (χ^2^*=*0.82, *p*=.365). For contact leg TA, there was neither a significant main effect of group (χ^2^*=*1.94, *p*=.164) nor trial (χ^2^*=*3.22, *p*=.073), nor a significant interaction between the two (χ^2^*=*0.40, *p*=.529). The treadmill speed covariate was not significant (χ^2^*=*2.24, *p*=.136).
